# Supplementary material for: Rapid On-Site Identification for Three Arcidae Species (Anadara kagoshimensis, Tegillarca granosa, and Anadara broughtonii) Using Ultrafast PCR Combined with Direct DNA Extraction
Source: Foods. 2022 Aug 14;11(16):2449. doi: 10.3390/foods11162449 (PMC9407576; doi:10.3390/foods11162449)
Supplement: Supplementary file 1 [file foods-11-02449-s001.zip › foods-1862433-supplementary.pdf]

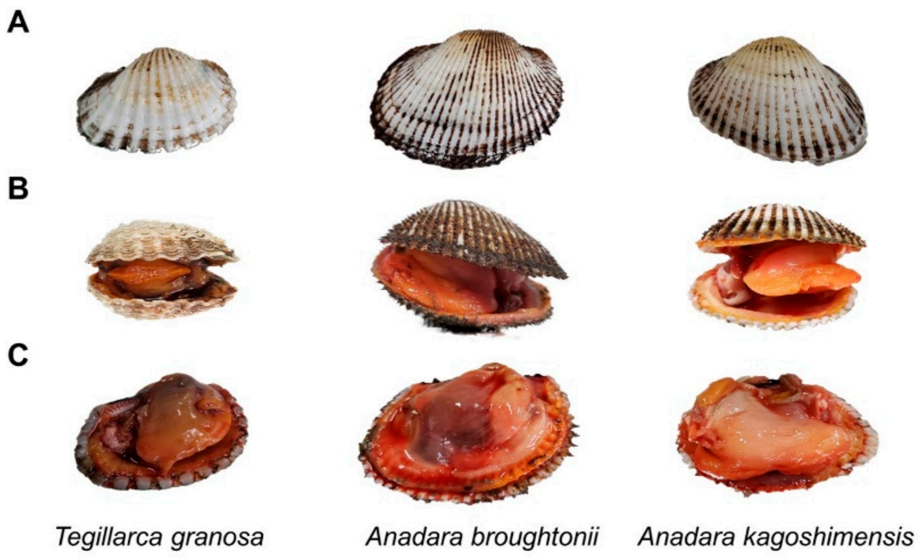

Figure S1. Morphological characteristics of granular ark (*Tegillarca granosa*), broughton's ribbed ark (*Anadara broughtonii*), and half-crenate ark (*Anadara kagoshimensis*). (A) shells, (B) shells with meat, and (C) ark shell meats.

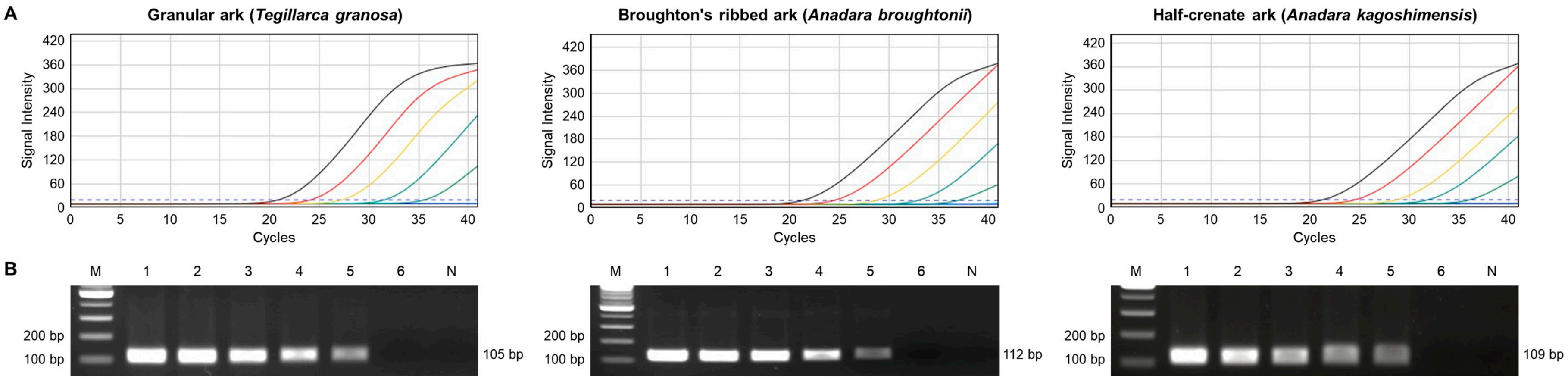

Figure S2. Sensitivity analysis of ultrafast PCR assay using serially diluted DNA (10 ng to 0.0001 ng). (A) Fluorescence of the amplified ark shell DNA was detected in real-time. (B) Electrophoresis of ultrafast PCR product: lane M, 100 bp DNA ladder; lane 1, 10 ng of target DNA; lane 2, 1 ng of target DNA; lane 3, 0.1 ng of target DNA; lane 4, 0.01 ng of target DNA; lane 5, 0.001 ng of target DNA; lane 6, 0.0001 ng of target DNA; lane N, non-template.
